# Barriers to Cervical Cancer Screening: A Systematic Review

**DOI:** 10.7759/cureus.65555

**Published:** 2024-07-28

**Authors:** Olugbenga Farajimakin

**Affiliations:** 1 Clinical Research, Faraj-Lab Medical Diagnostics and Research Center, Lagos, NGA

**Keywords:** screening and barriers, healthcare access, cultural factors, healthcare disparities, cervical cancer

## Abstract

Cervical cancer remains a significant global health concern, particularly in underserved populations. Despite the availability of effective screening methods, uptake remains suboptimal in many regions. This systematic review aims to synthesize the current evidence on barriers to cervical cancer screening across diverse populations and healthcare settings.

A comprehensive search of electronic databases was conducted to identify relevant studies published till June 2024. Studies examining barriers to cervical cancer screening in various populations were included. Data extraction and quality assessment were performed independently by two reviewers. A narrative synthesis approach was used to analyze and present the findings.

Seventeen studies met the inclusion criteria, encompassing a wide range of study designs and populations. Common barriers identified across studies included lack of knowledge and awareness, economic constraints, access issues, cultural and religious factors, fear and embarrassment, and distrust in healthcare systems. Population-specific barriers were observed among immigrant and ethnic minority women, individuals in low- and middle-income countries, indigenous women, and LGBQ women. Healthcare system factors, socioeconomic influences, psychological and individual factors, and interpersonal and community dynamics also played significant roles in screening participation.

This review highlights the complex and multifaceted nature of barriers to cervical cancer screening. Findings suggest that interventions to improve screening rates should be comprehensive, culturally sensitive, and tailored to specific population needs. Addressing both individual-level and systemic barriers is crucial for enhancing cervical cancer screening uptake globally.

## Introduction and background

Cervical cancer remains a significant global health concern, particularly among underserved populations. Despite being largely preventable through early detection and screening, it continues to be a leading cause of cancer-related deaths among women worldwide [1]. The World Health Organization (WHO) has called for the elimination of cervical cancer as a public health problem by 2030, emphasizing the critical role of screening and early detection in achieving this goal [2]. Underserved populations, including racial and ethnic minorities, low-income groups, rural residents, and individuals in low- and middle-income countries (LMICs), face disproportionate barriers to accessing cervical cancer screening services [3]. These disparities contribute to higher incidence and mortality rates among these populations [4]. Understanding the specific barriers that impede early detection and screening efforts is crucial for developing targeted interventions and policies to improve cervical cancer outcomes in underserved communities.

Recent studies have identified various obstacles to cervical cancer screening, including socioeconomic factors, cultural beliefs, healthcare system limitations, and individual-level barriers. However, a comprehensive synthesis of these barriers, particularly focusing on underserved populations across diverse global contexts, is lacking in the current literature [5,6]. The importance of addressing these barriers cannot be overstated. Cervical cancer is unique among cancers in that it is almost entirely preventable with proper screening and early intervention [7]. The development of cervical cancer is typically slow, progressing through detectable precancerous stages, which makes it ideal for screening programs [8]. However, the benefits of these advancements in medical knowledge and technology are not equitably distributed, with underserved populations often missing out on potentially life-saving screenings.

The disparity in cervical cancer incidence and mortality between served and underserved populations is stark. For instance, in the United States, African American women have a 30% higher cervical cancer incidence rate and a 70% higher mortality rate compared to white women [9]. Similarly, in many LMICs, where organized screening programs are often lacking, cervical cancer remains one of the leading causes of cancer death among women [10].

The barriers to cervical cancer screening are multifaceted and often interconnected. They can range from structural issues such as lack of healthcare access and affordability [11], to cultural factors including stigma and misconceptions about the screening process [6], to individual-level barriers such as fear of the procedure or lack of knowledge about its importance [12]. These barriers can be further compounded by factors such as immigration status, language barriers, and systemic racism in healthcare systems [13]. Moreover, the COVID-19 pandemic has exacerbated many of these existing barriers, with disruptions to healthcare services and increased reluctance to seek non-emergency medical care [14]. This has potentially set back progress in cervical cancer screening and early detection, particularly among already vulnerable populations.

The role of health literacy in cervical cancer screening cannot be overlooked. Studies have shown that women with lower health literacy are less likely to participate in screening programs and may have misconceptions about the purpose and process of screening [15]. This highlights the need for tailored education and communication strategies to reach underserved populations effectively. Another critical aspect is the influence of healthcare provider attitudes and practices on screening rates. Provider recommendation has been consistently identified as one of the strongest predictors of screening uptake [16]. However, studies have shown that providers may have biases or assumptions about certain populations that can affect their likelihood of recommending screening [17].

The advent of new screening technologies, such as HPV self-sampling, offers potential solutions to some traditional barriers. These methods can increase access to screening for women who face geographical, cultural, or personal barriers to clinic-based screening [18]. However, the implementation of these technologies in underserved populations faces its own set of challenges, including issues of acceptability, feasibility, and integration into existing healthcare systems. The intersectionality of barriers is another crucial consideration. Many women in underserved populations face multiple, overlapping barriers that compound their risk of missing out on screening. For example, a low-income immigrant woman might face financial barriers, language difficulties, cultural stigma, and lack of knowledge about the healthcare system simultaneously [5]. Understanding these intersecting barriers is essential for developing comprehensive, effective interventions.

Policy plays a significant role in addressing screening disparities. The implementation of national screening programs, insurance coverage for preventive services, and targeted outreach initiatives can significantly impact screening rates [19]. However, the effectiveness of these policies can vary widely between different contexts and populations, emphasizing the need for tailored, evidence-based approaches. As we move toward the WHO's goal of cervical cancer elimination, it is crucial to address these barriers systematically and comprehensively. This requires a nuanced understanding of how these barriers manifest in different contexts and for different populations. By synthesizing the existing literature on this topic, we can identify common themes, unique challenges, and successful interventions that can inform policy and practice.

This systematic review aims to provide a comprehensive overview of the barriers to cervical cancer screening in underserved populations, synthesizing evidence from diverse global contexts. By doing so, it seeks to inform the development of targeted interventions, guide policy decisions, and ultimately contribute to the reduction of cervical cancer disparities worldwide. To examine how these barriers differ across various underserved groups, such as racial and ethnic minorities, low-income populations, rural residents, and women in LMICs. To assess the relative impact of different barriers on screening uptake and adherence among underserved populations. To explore the intersectionality of multiple barriers and their compounded effects on cervical cancer screening behaviors. Moreover, to identify successful strategies and interventions that have effectively addressed these barriers in various contexts. To evaluate the impact of the COVID-19 pandemic on cervical cancer screening barriers and access for underserved populations. Additionally, it analyzes the role of health literacy and patient education in overcoming barriers to cervical cancer screening. To examine the influence of healthcare provider attitudes and practices on screening rates among underserved populations. Investigate the potential of innovative screening methods and technologies in overcoming traditional barriers to access. And finally, to provide evidence-based recommendations for policymakers, healthcare providers, and researchers to improve cervical cancer screening rates among underserved populations.

## Review

This systematic review followed the Preferred Reporting Items for Systematic Reviews and Meta-Analyses (PRISMA) guidelines. The review protocol was developed prior to conducting the search and analysis.

Research question

“What are the primary barriers to cervical cancer screening among underserved populations worldwide, and how do these barriers differ across various underserved groups?”

Patient, intervention, comparison, outcome (PICO) framework

Based on this PICO framework, the refined research question is “What are the primary barriers to cervical cancer screening (O) among underserved populations (P) worldwide, and how do these barriers differ across various underserved groups?” PICO along with the components is given in Table 1.

**Table 1:** PICO framework

PICO	Components
P (Population)	Underserved populations, including but not limited to: Racial and ethnic minorities, low-income individuals, rural residents, women in low- and middle-income countries (LMICs), immigrant women and other marginalized groups with lower access to healthcare.
I (Intervention/Issue)	Cervical cancer screening programs or opportunities.
C (Comparison)	Not applicable in this context, as we are not comparing interventions. However, we may compare barriers across different underserved groups.
O (Outcome)	Barriers to cervical cancer screening, including but not limited to: Socioeconomic barriers, cultural barriers, structural barriers, individual-level barriers, healthcare system-related barriers.

Eligibility criteria

Inclusion Criteria

Studies published till June 2024. Peer-reviewed articles in English. Primary research studies (quantitative, qualitative, or mixed methods). Studies focusing on barriers to cervical cancer screening. Studies involving underserved populations (e.g., racial/ethnic minorities, low-income groups, rural residents, women in LMICs). Studies reporting on at least one barrier to cervical cancer screening

Exclusion Criteria

Non-English language publications. Review articles, editorials, commentaries, or conference abstracts. Studies do not specifically address barriers to cervical cancer screening. Studies focus solely on general populations without emphasis on underserved groups. Studies reporting only on cervical cancer incidence or treatment without addressing screening barriers.

Protocol

The protocol followed the PRISMA guidelines and outlined the research question, search strategy, inclusion and exclusion criteria, data extraction methods, and analysis plan.

Information sources and search strategy

We conducted a comprehensive search of electronic databases including PubMed, EMBASE, CINAHL, and Scopus. The search strategy was developed in consultation with a medical librarian and included a combination of MeSH terms and keywords related to cervical cancer screening, barriers, and underserved populations. The search terms were adapted for each database while maintaining consistency in the core concepts. The PICO framework with corresponding MeSH terms is given in Table 2.

**Table 2:** PICO framework presented in a table with corresponding MeSH terms

Component	MeSH Terms
P (Population)	"VulnerablePopulations" (Mesh), "Minority Groups" (Mesh), "Rural Population" (Mesh), "Emigrants and Immigrants" (Mesh), "Poverty" (Mesh), "Developing Countries" (Mesh)
I (Intervention/Issue)	"Mass Screening" (Mesh), "Early Detection of Cancer" (Mesh), "Uterine Cervical Neoplasms/diagnosis" (Mesh)
C (Comparison)	N/A
O (Outcome)	"Health Services Accessibility" (Mesh), "Healthcare Disparities" (Mesh), "Socioeconomic Factors" (Mesh), "Cultural Characteristics" (Mesh), "Health Knowledge, Attitudes, Practice" (Mesh)

The search was limited to articles published till June 2024. Additional relevant studies were identified through hand-searching of reference lists of included articles and key reviews in the field.

Study Selection Process

**Figure 1:**
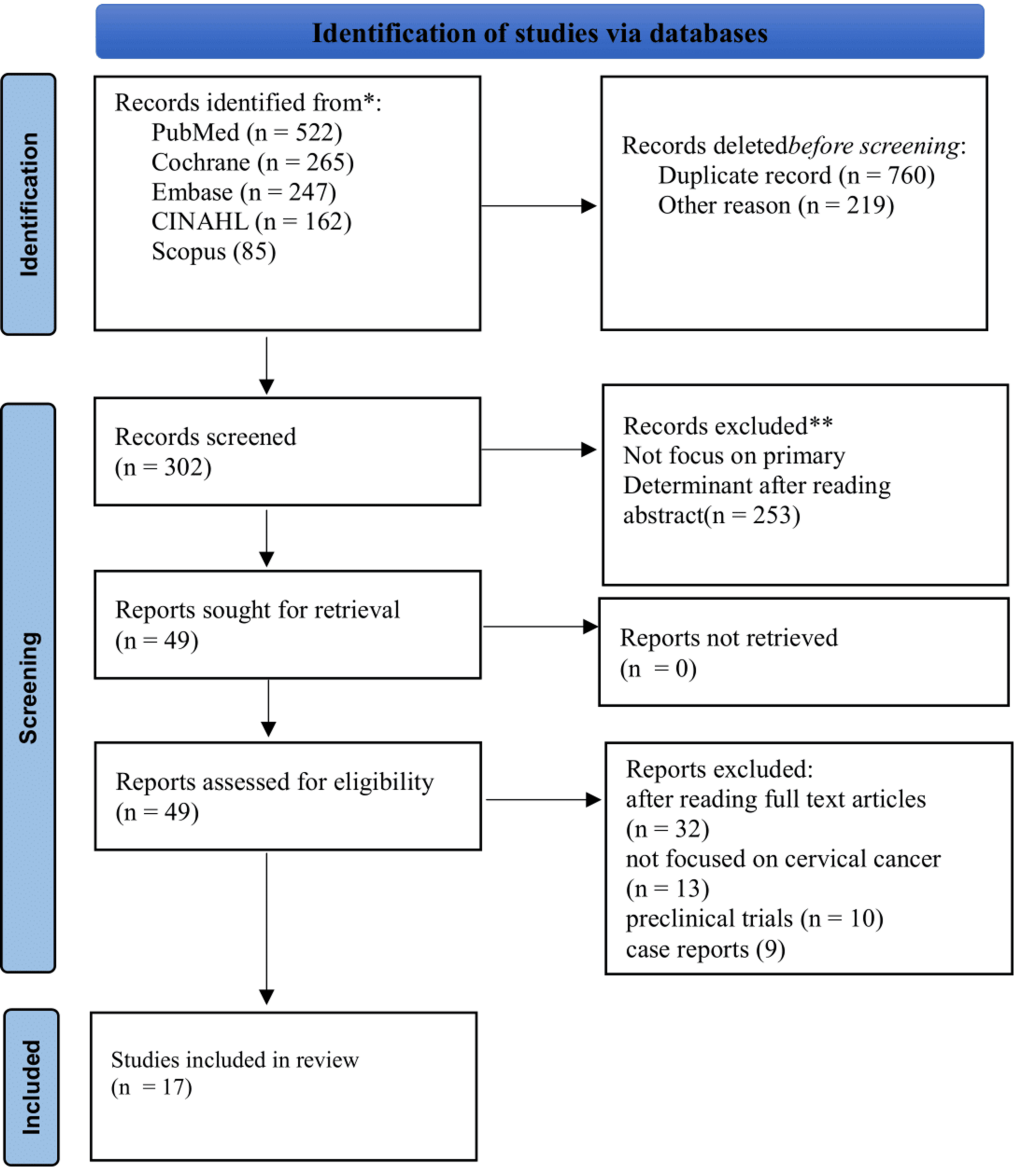
PRISMA flow chart

Two independent reviewers screened titles and abstracts, followed by a full-text review of potentially eligible studies. This process guaranteed a systematic and unbiased selection of relevant studies for the current review. The total number of identified records was 1,281, including PubMed/MEDLINE (522), Cochrane Library (265), EMBASE (247), CINAHL (162), and Scopus (85). Figure 1 explains the details of the PRISMA flow chart and the identification of the studies.

Data Collection Process

Data extraction was performed using a standardized form, capturing information on study characteristics, population, barriers identified, and any interventions or recommendations provided.

Quality of Studies

The quality of included studies was assessed using appropriate tools for different study designs, such as the Newcastle-Ottawa Scale for observational studies and the Critical Appraisal Skills Program (CASP) checklist for qualitative studies [20,21].

The NOS scale uses a star system, with a maximum of nine stars. Studies are judged on eight items categorized into the three perspectives mentioned above. A study can receive a maximum of one star for each numbered item within the Selection and Outcome categories, and a maximum of two stars can be given for Comparability [20].

In CASP, each question is answered with “Yes,” “No,” or “Can't Tell.” While there is no numerical scoring system, the answers provide a structured way to assess the overall quality and rigor of a qualitative study [22].

Risk of Bias Assessment

We used the Cochrane Risk of Bias 2.0 tool (London, England) for randomized trials that covered five domains. Each domain is assessed as “Low risk,” “Some concerns,” or “High risk.” The overall risk of bias is then determined based on the domain-level judgments.

The ROBINS-I tool for non-randomized studies assesses seven domains. Each domain is assessed as “Low risk,” “Moderate risk,” “Serious risk,” or “Critical risk” of bias. The overall risk of bias is determined based on these domain-level assessments.

Results

Characteristics of the Included Studies

Following are the systematically reviewed details of the included studies. Table 3 explains the functional details of the studies.

**Table 3:** Characteristics of the included studies

Author (Year)	Region	Study Design	Sample Size	Population	Main Barriers Identified
Let al. (2009) [22]	Developed countries	Systematic review	19 studies	General population	Varied by study; no consistent pattern
Marlow et al. (2015) [23]	England	Structured interviews	720	BAME women	Low perceived risk, belief screening unnecessary without symptoms, scheduling difficulties
Rosser et al. (2015) [24]	Kenya	Provider survey	106	Healthcare staff	Staffing shortages, lack of trained staff, insufficient space, supply issues
Gele et al. (2017) [25]	Norway	Qualitative focus groups	35	Somali and Pakistani immigrants	Lack of understanding, stigma, cultural beliefs, lack of trust in healthcare system
Lim et al. (2017) [26]	Sub-Saharan Africa	Systematic review	Not specified	General population	Fear, low awareness, embarrassment, lack of spousal support, cost, access issues
Shrestha et al. (2017) [27]	Nepal	Cross-sectional survey	96	Women attending gynecology OPD	Inadequate knowledge, lack of awareness
Ferdous et al. (2018) [13]	Canada	Systematic review	Not specified	Immigrant women	Economic, cultural, language, healthcare system, knowledge, and individual-level barriers
Guillaume et al. (2020) [28]	LMICs	Systematic review	Not specified	Women living with HIV	Lack of knowledge, healthcare system barriers
Kandasamy et al. (2022) [29]	Various	Systematic review	9 studies	Indigenous women	Systemic and cultural barriers
Nilima et al. (2022) [30]	India	Secondary data analysis	Not specified	General population	Age, education, awareness, socioeconomic factors
Zhang et al. (2023) [31]	China	Cross-sectional survey	4,518	General population	Limited knowledge, low screening coverage
Rosato et al. (2023) [32]	Various	Systematic review & meta-analysis	18 studies	Migrant women	Lower participation rates compared to native women
Srinath et al. (2023) [33]	LMICs	Systematic review	67 studies	General population	Lack of awareness, cost, distance, embarrassment, cultural factors
Afsah et al. (2023) [34]	Various	Scoping review	Not specified	Immigrant Muslim women	Sociodemographic, economic, language, cognitive, emotional, and religious barriers
Emerson et al. (2024) [35]	USA	Qualitative interviews	20	Women with criminal-legal system involvement	Interpersonal communication issues, logistical barriers
Mantula et al. (2024) [36]	Africa	Mixed-methods review	24 studies	General population	Poor access, lack of awareness, socio-cultural influences
Spencer et al. (2024) [37]	USA	Survey	753	Low-income women	Lack of insurance, cost, forgetfulness, transportation (higher in LGBQ women)

Synthesis of Results

Data synthesis was conducted using a narrative approach, given the heterogeneity of study designs and outcomes. We categorized barriers into themes and sub-themes, analyzing patterns and differences across various underserved populations and contexts.

Common Barriers Across Studies

Common barriers across studies included lack of knowledge and awareness about cervical cancer and screening, economic barriers such as cost and lack of insurance, access issues related to transportation and distance to healthcare facilities, cultural and religious factors, fear and embarrassment, and lack of trust in healthcare systems [13,25,27,34,38].

Population-Specific Barriers

Population-specific barriers were identified for various groups. Immigrant and ethnic minority women faced additional challenges related to language, cultural beliefs, and integration into new healthcare systems [13,25,26,32,34]. Women in LMICs often encounter structural barriers such as a lack of resources and trained personnel [24,28,36]. Indigenous women faced systemic and historical barriers [29], while LGBQ women experienced unique challenges such as higher rates of forgetfulness and transportation issues [37].

Healthcare System Factors

Healthcare system factors played a significant role, with studies reporting staffing shortages and lack of trained personnel, especially in LMICs, insufficient resources and supplies, and the need for better communication and culturally sensitive care [24,28,36].

Socioeconomic Influences

Socioeconomic influences were consistently associated with screening uptake. Education level and socioeconomic status were found to be important factors, while cost and lack of insurance were significant barriers, particularly in countries without universal healthcare [30,37].

Psychological and Individual Factors

Psychological and individual factors included a low perceived risk of cervical cancer, the belief that screening is unnecessary without symptoms, and competing health priorities, especially for women with complex health needs [6,31].

Interpersonal and Community Factors

Interpersonal and community factors such as lack of spousal or family support, community stigma around cancer and screening, and the importance of social networks and community education were also highlighted [25,26].

Variability Across Regions

While many barriers were common across studies, their relative importance varied by region, culture, and healthcare system [22].

Special Populations

Special populations, such as women with HIV, those involved in the criminal legal system, and other marginalized groups, faced compounded barriers [28,35]. This synthesis highlights the multifaceted nature of barriers to cervical cancer screening, emphasizing the need for comprehensive, culturally sensitive, and population-specific approaches to improve screening rates globally. Interventions should address not only individual-level factors but also systemic, cultural, and socioeconomic barriers to be effective.

Identification and Categorization of Barriers

The review highlighted the complex interplay between different types of barriers. For example, an immigrant woman might face language barriers (cultural) while also struggling with the cost of screening (socioeconomic) and unfamiliarity with the healthcare system (structural). A woman in a rural LMIC might face distance to healthcare facilities (structural), coupled with cultural taboos around cancer screening (cultural) and limited personal resources (socioeconomic). This intersectionality underscores the need for comprehensive, multi-faceted approaches to improving screening rates (Table 4).

**Table 4:** Categories of barriers to cervical cancer screening

Category	Specific barriers
Socioeconomic	Cost of screening, Lack of health insurance, Low education levels, Poverty
Cultural	Stigma associated with cancer and screening, Religious beliefs, Lack of spousal support, Cultural taboos around sexual health
Structural	Limited access to healthcare facilities, Lack of trained personnel, Insufficient resources and equipment, Long waiting times
Individual	Lack of knowledge about cervical cancer and screening, Low perceived risk, Fear of the screening procedure, Embarrassment, Lack of time

Difference Across Underserved Groups

The relative impact of barriers: While the review did not provide quantitative measures of impact, certain barriers were consistently identified as significant across multiple studies, including lack of knowledge and awareness, cost and lack of insurance, access issues (distance, availability of services), and intersectionality of barriers. These factors appear to have the most substantial impact on screening rates across various populations and contexts. Table 5 explains the details regarding the impact of barriers.

**Table 5:** Barriers faced by different underserved groups LMICs: Low- and middle-income countries

Group	Key barriers
Racial and ethnic minorities	Language barriers, Cultural conflicts, Historical mistrust
Low-income populations	Cost, Lack of insurance, Competing priorities
Rural residents	Geographic distance, Limited specialized services
Women in LMICs	Resource scarcity, Limited awareness, Socio-cultural influences

Successful strategies and interventions: The review consistently emphasized the importance of knowledge and awareness. While not extensively covered, some promising strategies and future interventions emerged are given in Table 6.

**Table 6:** Promising strategies and interventions

Strategy	Description
Community-based education	Programs tailored to local needs and cultures
Culturally sensitive care	Healthcare delivery respecting diverse beliefs and practices
Policy changes	Addressing systemic barriers through legislation
Mobile screening units	Improving access in rural and underserved areas
Integrated health services	Combining cervical cancer screening with other health services

Recommended research areas: The review identified a gap in understanding the impact of pandemic and other scenarios on cervical cancer screening. Future research should explore the given areas mentioned in Table 7.

**Table 7:** Areas for future research

Research Area	Focus
COVID-19 impact	Screening rate changes, new barriers, telehealth effectiveness
Health literacy	Culturally appropriate materials, diverse communication channels
Provider influence	Attitudes, gender impact, communication strategies
Innovative methods	HPV self-sampling, mobile technologies, AI-assisted screening

Based on the synthesis, key recommendations are given in Table 8. These visual representations provide a clear overview of the key findings from the systematic review, highlighting the barriers faced by different groups, promising strategies, areas for future research, and evidence-based recommendations.

**Table 8:** Evidence-based recommendations

Recommendation	Description
Education programs	Comprehensive, culturally sensitive awareness initiatives
Economic barrier reduction	Policy changes and financial support mechanisms
Healthcare infrastructure	Improved facilities and provider training, especially in LMICs
Targeted interventions	Tailored approaches for specific underserved populations
Service integration	Combining cervical cancer screening with other health services

Discussion

This systematic review provides a comprehensive analysis of barriers to cervical cancer screening among underserved populations worldwide. The findings align with and expand upon previous research in this field, highlighting the complex interplay of socioeconomic, cultural, structural, and individual factors that contribute to low screening rates.

Socioeconomic barriers, particularly cost and lack of insurance, were consistently identified as significant obstacles across multiple studies [30,33,37]. This aligns with the work of Adunlin et al., who found that financial constraints were a primary barrier to cancer screening in LMICs [39]. Our review further emphasizes that these economic barriers are not limited to LMICs but also affect underserved populations in high-income countries, particularly those without universal healthcare systems.

Cultural barriers, including stigma, religious beliefs, and lack of spousal support, emerged as crucial factors influencing screening behavior, especially among immigrant and ethnic minority women [29,34]. This finding is consistent with the work of Marlow et al. and Gele et al., who identified similar cultural barriers among ethnic minority women in the UK and Norway, respectively [25]. Our review extends these findings by highlighting the intersectionality of cultural barriers with other factors, such as economic constraints and healthcare system distrust.

Structural barriers, including limited access to healthcare facilities and lack of trained personnel, were particularly prominent in studies from LMICs and rural areas [28,37]. This aligns with the systematic review by Lim and Ojo, which identified similar structural barriers in Sub-Saharan Africa [26]. Our review adds to this knowledge by demonstrating that these structural barriers persist across various geographical and economic contexts, albeit to different degrees.

Individual-level barriers, such as lack of knowledge, low perceived risk, and fear, were consistently reported across studies [33,35,40]. This corroborates the findings of Ferdous et al., who identified similar individual-level barriers among immigrant women in Canada [13]. Our review extends this understanding by demonstrating how these individual factors interact with broader structural and cultural barriers.

The role of healthcare providers in influencing screening rates, as highlighted in our review, aligns with the work of Rosser et al. in Kenya [24]. However, our findings suggest that this influence extends beyond LMICs and is relevant in various healthcare contexts, emphasizing the need for provider education and support.

Limitations of the study

While our review didn't extensively cover the impact of COVID-19 on screening barriers, emerging research suggests that the pandemic has exacerbated existing disparities and created new challenges for cervical cancer screening [41]. This highlights an important area for future research.

The potential of innovative screening methods in overcoming traditional barriers, though not extensively covered in our review, has been explored in other studies. For instance, Yeh et al. discussed the promise of HPV self-sampling in increasing screening uptake among underserved populations [42]. This represents an important direction for future research and intervention development.

Future recommendation

Building upon the findings of this systematic review, several important directions for future research emerge. Firstly, there is a pressing need to evaluate the long-term impact of the COVID-19 pandemic on cervical cancer screening disparities, as the pandemic has likely exacerbated existing barriers and created new challenges for underserved populations. Secondly, researchers should focus on developing and testing innovative, culturally tailored interventions that address multiple barriers simultaneously, as our review has highlighted the complex interplay of various factors affecting screening uptake. The potential of new technologies, such as HPV self-sampling and artificial intelligence-assisted screening, in overcoming traditional barriers deserves thorough investigation, particularly in resource-limited settings.

Future studies should also explore the intersectionality of multiple barriers and their cumulative effects on screening behaviors, as this understanding could inform more effective, targeted interventions. Longitudinal studies assessing the long-term effectiveness of interventions in improving screening rates among underserved populations are crucial to ensure sustainable improvements in cervical cancer prevention. Additionally, examining the role of community-based participatory research in developing effective, culturally appropriate screening programs could provide valuable insights into engaging underserved communities. Finally, investigating the impact of policy changes, such as implementing universal healthcare coverage, on screening disparities could inform broader systemic interventions to improve cervical cancer screening rates. By pursuing these research directions, we can continue to build a more comprehensive understanding of barriers to cervical cancer screening and develop more effective strategies to address them, ultimately working toward reducing global disparities in cervical cancer outcomes.

## Conclusions

This systematic review provides a comprehensive overview of the multifaceted barriers to cervical cancer screening among underserved populations worldwide. The findings underscore the complex interplay of socioeconomic, cultural, structural, and individual factors that contribute to low screening rates. While many barriers are common across different contexts, their relative importance and specific manifestations vary by population and setting.
